# Bullying, State Policy, and Mental Health Symptoms in Gender-Diverse Youths

**DOI:** 10.1001/jamanetworkopen.2026.8104

**Published:** 2026-04-21

**Authors:** Dylan E. Hughes, Sarah L. Zapetis, Arianna Mordy, Daisy Lopez, Vanessa Calderon, Laura Adery, Rachel Martino, Sarah E. Chang, Lucina Q. Uddin, Carlos Cardenas-Iniguez, Richard T. Lebeau, Natalia Ramos, Lauren C. Ng, Katherine H. Karlsgodt, Carrie E. Bearden

**Affiliations:** 1 Department of Psychology, University of California, Los Angeles; 2 Department of Psychology, University of Southern California, Los Angeles; 3 Department of Psychiatry and Biobehavioral Sciences, Semel Institute for Neuroscience and Human Behavior, University of California, Los Angeles; 4 Department of Cognitive Science, University of California, San Diego; 5 Department of Psychology, Harvard University, Cambridge, Massachusetts; 6 Department of Population and Public Health Sciences, Keck School of Medicine, University of Southern California, Los Angeles; 7 Department of Psychology, California State University, Fullerton

## Abstract

**Importance:**

As the percentage of young people in the US identifying as transgender and gender-diverse increases, more youths face identity-linked social and structural stigma and discrimination. Little is known about the impact of stigma on psychotic-like experiences in gender-diverse youths.

**Objective:**

To assess the association of being bullied and state policies with psychotic-like experiences among gender-diverse youths.

**Design, Setting, and Participants:**

In this prospective cohort study, cross-sectional and longitudinal analyses were conducted between 2023 and 2025 using data collected between January 1, 2017, and December 31, 2022, across 17 states. The Adolescent Brain Cognitive Development (ABCD) Study is a US population–based, longitudinal study that follows and deeply phenotypes youths from the age of 9 to 18 years. Cross-sectional analyses included data from 8463 participants collected between January 1, 2019, and December 31, 2022. Longitudinal analyses included 4200 participants with data collected across 5 waves between January 1, 2017, and December 31, 2022. Statistical analysis was performed from January 1, 2023, to December 31, 2025.

**Exposures:**

Self-reported frequency of being bullied and data on annual state policies related to gender identity.

**Main Outcomes and Measures:**

Self-reported PLEs and associated distress, measured by the Prodromal Questionnaire–Brief Child Version.

**Results:**

The study sample included 8463 youths (mean [SD] age, 12.9 [0.6] years; 4490 [53.1%] assigned male at birth). Based on a dimensional measure of gender, 531 youths (6.3%) were identified as most gender diverse (ie, least congruent with birth-assigned sex) and 6867 (81.1%) as least gender diverse (ie, most congruent with birth-assigned sex). Rates of being bullied and PLEs were significantly elevated in the most vs least gender-diverse group, with bullying partially mediating the difference in PLEs (indirect association = 0.15, *P* < .001; direct association = 0.63, *P* < .001). Moreover, the persistence of unsupportive legislation across 4 years was associated with significantly greater increases in PLEs over time in gender-diverse youth (interaction β = 0.31; 95% CI, 0.21-0.42; *P* < .001).

**Conclusions and Relevance:**

In this cohort study, being bullied and unsupportive legislation were associated with greater and increasing rates of PLEs in gender-diverse youths. This study’s findings suggest that mental health in gender-diverse youths could benefit from supportive environments and policies that reduce bullying and avoid perpetuating stigma.

## Key Points

**Question:**

Are bullying and state policies related to gender identity associated with subclinical psychotic-like experiences (PLEs) in gender-diverse youths in the US?

**Finding:**

In this large, nationally representative cohort study of 8463 youths aged 9 to 13 years, self-reported bullying was associated with greater rates of PLEs in gender-diverse youths. Across 4 years, consistently unsupportive policy related to gender identity was associated with increasing PLEs in gender-diverse youths.

**Meaning:**

These findings suggest that social stigma, including bullying and unsupportive legislation related to gender identity, plays an important role in shaping PLEs and their trajectories in gender-diverse youths, highlighting the need for supportive environments and policies.

## Teaser

This cohort study examines whether interpersonal and structural distal stressors are associated with psychotic-like experiences among gender-diverse youths aged 9 to 13 years.

## Introduction

Between 2017 and 2022, the percentage of adolescents (ages 13-17 years) identifying as transgender and gender diverse (TGD) in the US doubled from 0.73%^1^ to 1.43%.^2^ As gender is increasingly reported on a spectrum,^3,4^ these numbers may underestimate the proportion of the US adolescent population whose gender identity is dimensionally incongruent with their birth-assigned sex (eg, a birth-assigned male who does not feel entirely like a boy). As gender becomes increasingly nuanced, there is an increasing need to understand the mechanisms underlying the disproportionate prevalence of mental health problems among TGD youths^5,6,7^ and whether dimensionally incongruent youths are at risk for the same effects.

Psychotic spectrum symptoms in particular are overrepresented in TGD individuals. TGD adults are 3.0 to 49.7 times more likely to have a schizophrenia spectrum disorder diagnosis than cisgender adults.^8^ In TGD youths, estimates for the prevalence of psychotic-like experiences (PLEs)—psychotic spectrum symptoms that may be accompanied by distress and/or indicate risk for clinically significant psychosis^9^—are sparse. Some small studies have found that TGD youths and young adults (ages 12-28 years) are overrepresented in samples at high risk for clinically significant psychosis.^10^ Nevertheless, the etiology of psychosis across its continuum in TGD youths remains understudied.^11^

The minority stress model^12^ provides a framework to examine the mechanisms through which minoritized groups, including TGD youths, experience higher rates of mental health problems, such as psychosis. The model proposes that long-term exposure to external (ie, distal) stressors, such as discrimination and rejection, shapes negative attitudes toward one’s identity (ie, proximal stressors), which in turn may lead to mental health challenges.^11,12^ In line with this framework, TGD youths experience high rates of, and are more at risk for peer bullying, discrimination, and other interpersonal stressors.^6,13,14^

Structural stigma (ie, public attitudes, societal conditions, and/or policies that explicitly or implicitly impose barriers to the well-being of minoritized groups) is another form of distal stress that can negatively impact mental health in minoritized individuals.^15,16,17^ In TGD youths in the US, supportive policies (eg, antidiscrimination laws in housing, school, and public spaces) have been shown to protect against mental health problems.^18,19^ Unsupportive policies (eg, “bathroom laws” that block transgender people from using public spaces aligned with their gender identity) are associated with poorer mental health.^20^ Alarmingly, one US study showed that, after the enactment of unsupportive laws between 2018 and 2022, the incidence of suicide attempts among TGD youths increased by 7% to 72%.^20^ This association is particularly concerning given the proliferation of legislation targeting lesbian, gay, bisexual, transgender, and queer/questioning (LGBTQ+) US residents; as of June 2025, 588 anti-LGBTQ+ bills had been introduced across the US in 2025, twice as many as were introduced throughout the entire year of 2022.^21^

Hypervigilance and paranoid ideation are core features of psychosis^22^ and may be engendered by exposure to the unique interpersonal stressors and structural stigma experienced by minoritized groups.^23^ Although interpersonal stressors, such as bullying, partially explain high rates of PLEs in youths with diverse expressions of gender,^24^ it is unclear whether this is evident in youths with marginally incongruent gender identities. To our knowledge, no studies have reported on the effects of policy on PLEs in TGD youths.

In this study, we explore the association of 2 distinct forms of distal stressors—interpersonal (ie, bullying) and structural (ie, a lack of supportive state policies related to gender identity)—with PLEs in a large youth dataset collected across 17 states. We hypothesized that (1) bullying would mediate the association between gender diversity and PLEs, (2) the association of bullying with PLEs would be stronger in the most gender-diverse youths compared with non–gender-diverse youths, and (3) the absence of supportive legislation would be associated with more PLEs in gender-diverse youths.

## Methods

### Data

The analytic sample was derived from 11 868 youths enrolled in the Adolescent Brain Cognitive Development (ABCD) Study (data release 5.1), a population-based, prospective, longitudinal study across 21 US sites (17 states) following up youths annually from the ages of 9 to 18 years. Recruitment for the ABCD Study occurred between September 2016 and August 2018, which sought to sample participants from varying backgrounds to capture the socioeconomic as well as racial and ethnic diversity of the US. At all ABCD Study sites, written informed consent was obtained from parents and assent from children. Each ABCD Study site’s institutional review board provided local approval, with centralized institutional review board approval at the University of California, San Diego. State-level legislative data were provided by the Movement Advancement Project (MAP),^25^ an organization that collates data related to LGTBQ+ rights in the US. Cross-sectional analyses were performed on the year 3 data (modal interview year 2020), the largest quantity of data (n = 10 123) at the oldest age (mean [SD] age, 12.9 [0.6] years). Longitudinal analyses were conducted across years 1 (n = 11 220; mean [SD] age, 10.9 [0.6] years; modal year 2018) through 4 (n = 4688; mean [SD] age, 14.1 [0.7] years; modal year 2021). The current study followed the Strengthening the Reporting of Observational Studies in Epidemiology (STROBE) reporting guideline for cohort studies.

### Gender Diversity

**Figure 1.**
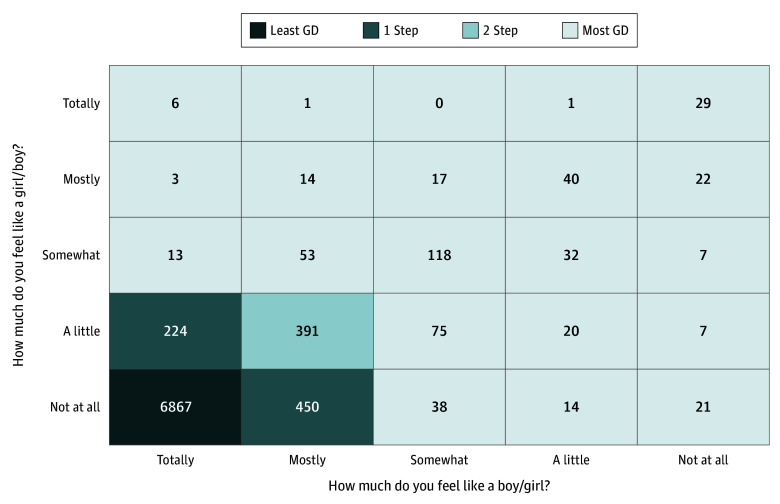
Matrix Graph of the Operationalization of Gender Diversity Data From the 2 Felt-Gender Questions at Year 3 The cross-sectional analyses were conducted in year 3. Participants who answered that they felt totally like the gender associated with their birth-assigned sex and not at all like the other gender were categorized in the least gender-diverse (GD) group (n = 6867). Participants who responded that they felt somewhat, a little, or not at all like their birth-assigned gender and somewhat, mostly, or totally like the other gender were categorized in the most GD group (n = 531). One step refers to participants who endorse a minimally GD experience of gender (eg, an individual assigned female at birth endorsing feeling “not at all” like a boy and “mostly” like a girl). Two step refers to participants who endorse an experience of gender that is minimally more diverse than 1 step (eg, an individual assigned male at birth endorsing feeling “mostly” like a boy and “a little” like a girl). Adapted from Potter et al.^4^

Gender diversity was operationalized using responses to 2 questions on a gender questionnaire constructed by the ABCD Study to measure felt-gender^4^: “How much do you feel like a boy?” and “How much do you feel like a girl?” with response options on a 5-point Likert scale, with 1 indicating “Not at all” and 5 indicating “Totally.” At each time point, cutoff points were applied to the scores to create 4 groups at each time point, ranging from the least gender diverse (ie, felt-gender most congruent with birth-assigned sex) to most gender diverse (ie, felt-gender least congruent with birth-assigned sex) (Figure 1; eTable 1 in Supplement 1). Unless otherwise noted, the findings of group 4 (most gender diverse) vs group 1 (least gender diverse [referent]) were the primary outcomes interpreted.

### Psychotic-Like Experiences

PLEs were measured via the Prodromal Questionnaire–Brief Child Version (PQ-BC), a 21-item, developmentally appropriate, self-report measure of PLEs and associated distress, using a 5-point Likert scale. The distress-weighted sum of the responses to all items was used in analyses, as done previously,^26,27^ resulting in possible scores between 0 and 126 (see eMethods in Supplement 1).

### Broad Mental Health Problems

The Brief Problem Monitor (BPM) total T score was used as a measure of general psychopathology. The BPM is a 19-item self-report measure that assesses mental health problems across multiple domains, where the total score is the scaled sum of all items.^28^

### Bullying Experiences and Perpetration

Participants reported on the frequency of bullying experiences via a 9-item questionnaire, the Peer Experiences Questionnaire (PEQ), with each item endorsed on a scale of 1 (never) to 5 (a few times a week). The PEQ consists of 2 versions: one inquiring about being bullied and the other about perpetrating bullying. Because the sum of all 9 bullied items results in a range of 9 to 45, the score was first adjusted to enhance interpretability by subtracting 9 from all values. The resultant PEQ-Vic score (range, 0-36) was used for primary analyses with greater scores representing greater rates of being bullied. The perpetration scale was adjusted similarly and included as a covariate in sensitivity analyses because being bullied is correlated with perpetration.^29^

### State Policies Related to Gender Identity or Expression

**Figure 2.**
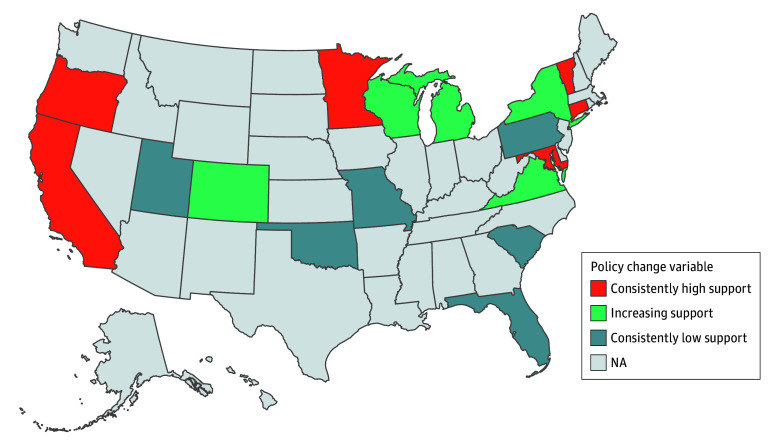
US Map Showing Geographic Representation of the Primary Change (Longitudinal) Policy Variable Analyzed in the Current Analyses Each participant was assigned a value, categorized into 1 of 3 groups, based on the change in the policy tally (see eFigure 3 in Supplement 1 for yearly tallies). Importantly, the longitudinal policy variable is at the person level not the state level. However, overwhelmingly, participants in the same state had the same change policy variable. Missouri and Connecticut were the only 2 states where participants’ change policy variable differed based on their baseline participation year (eFigure 2 in the Supplement 1). Consistently high support represents states with relatively high support across 4 years; consistently low support, states with relatively low support across 4 years; and increasing support, states that increased from relatively low to relatively high support across 4 years. NA indicates not applicable.

Tallies of enacted protective and antitransgender legislation for all US states across all years of data collection (January 1, 2017, to December 31, 2022) were provided by MAP,^25^ which assigns each state a score based on the number of enacted laws related to LGTBQ+ rights (see eTable 2 in Supplement 1 for examples). In the current analysis, the gender identity–related MAP tallies were analyzed, representing tallies of laws that “explicitly address or impact gender identity and/or expression.”^25^ Tallies were converted to a proportion based on the maximum tally for a given year. Thus, each participant was assigned a value, wherein a score closer to 1 represents a participant residing in a state with more supportive gender identity policies, and a score closer to 0 represents a state with less supportive policies. For longitudinal analyses, because most state scores varied minimally across the 4 years, states were categorized as having consistently high (ie, scores >0.5 across time), consistently low (scores <0.5), or increasingly supportive policies (Figure 2; eFigures 1-3 and eMethods in Supplement 1).

### Statistical Analysis

All statistical analyses were conducted with R statistical software, version 4.3.1 (R Foundation for Statistical Computing)^30^ between January 1, 2023, and December 31, 2025. Unequal representation of categorical variables and differences in means of continuous variables across the 4 levels of the gender diversity variable were tested for statistical significance with χ^2^ tests and analyses of variance (Table). All main analyses modeling differences in (continuous) mental health outcomes by gender diversity were conducted with linear mixed-effects regression (lme4 package). Notably, the distribution of PQ-BC scores is zero-inflated and positively skewed. As such, zero-inflated γ-distribution general linear models (hurdle models; glmmTB package in R) were additionally conducted to confirm that main results were not substantially affected by a violation of the assumptions of linear regression. Results were largely consistent; as such, to facilitate interpretation and comparison to other ABCD Study analyses of the PQ-BC measure,^26,27^ main results were reported for linear mixed-effects regression models. Unless otherwise stated, all reported coefficients were standardized and thus represent a β change in Y for each SD increase in X.

**Table.** Demographic Characteristics at Year 3 Data Collection

Characteristic	Least GD (n = 6867)	1 Step (n = 674)	2 Step (n = 391)	Most GD (n = 531)	*P* value
Age, mean (SD), y	12.9 (0.6)	12.8 (0.6)	12.8 (0.6)	12.9 (0.6)	<.001
Birth-assigned sex, No. (%)					
Female	2772 (40.4)	451 (66.9)	310 (79.3)	440 (82.9)	<.001
Male	4095 (59.6)	223 (33.1)	81 (20.7)	91 (17.1)	
Pubertal development, mean (SD), y	2.4 (0.7)	2.7 (0.7)	2.8 (0.6)	2.9 (0.6)	<.001
Race, No. (%)					
American Indian, Alaska Native, or non-Hispanic Pacific Islander	38 (0.6)	5 (0.7)	3 (0.8)	4 (0.8)	<.001
Asian	151 (2.2)	16 (2.4)	8 (2.1)	4 (0.8)	
Black	793 (11.7)	96 (14.3)	41 (10.5)	88 (16.8)	
White	4776 (70.2)	444 (66.4)	262 (67.2)	318 (60.6)	
Multiracial	793 (11.7)	82 (12.3)	62 (15.9)	91 (17.3)	
Other^a^	252 (3.7)	26 (3.9)	14 (3.6)	20 (3.8)	
Ethnicity, No. (%)					
Non-Hispanic	5529 (81.4)	534 (80.4)	315 (82.0)	403 (76.8)	.06
Hispanic	1261 (18.6)	130 (19.6)	69 (18.0)	122 (23.2)	
Parental educational level, mean (SD), y	17.5 (2.4)	17.2 (2.7)	17.5 (2.3)	16.9 (2.4)	<.001
Family income score, mean (SD)^b^	7.6 (2.2)	7.4 (2.3)	7.5 (2.2)	7.0 (2.5)	<.001
Severity of distressing psychotic-like experiences score, mean (SD)	2.0 (5.2)	4.3 (8.1)	5.9 (9.1)	7.5 (11.0)	<.001
Frequency of being bullied score, mean (SD)^c^	2.8 (3.6)	3.3 (3.9)	4.3 (4.9)	4.6 (5.1)	<.001
Frequency of perpetrating bullying score, mean (SD)^c^	1.2 (1.9)	1.4 (2.2)	1.5 (2.2)	1.5 (2.3)	<.001
Broad mental health problems T score, mean (SD)^d^	53.3 (5.2)	56.0 (6.5)	58.1 (6.8)	58.7 (7.2)	<.001

Abbreviation: GD, gender diverse.

^a^ No additional information provided for other race.

^b^ Estimated combined family income was reported by parents according to the following scale: 1, less than $5000; 2, $5000 to $11 999; 3, $12 000 to $15 999; 4, $16 000 to $24 999; 5, $25 000 to $34 999; 6, $35 000 to $49 999; 7, $50 000 to $74 999; 8, $100 000 to $199 999; and 10, $200 000 and greater.

^c^ A description of the score is given in the Bullying Experiences and Perpetration subsection of the Methods section.

^d^ A description of the score is given in the Broad Mental Health Problems subsection of the Methods section.

For all cross-sectional models, only participants with complete data for all primary variables (ie, PQ-BC, PEQ-Vic, gender diversity, state policy, and covariates) were retained for analysis (n = 8463). See eTable 3 in Supplement 1 for observed differences between the analyzed sample and omitted sample. Cross-sectional analyses included random intercepts for families nested within sites (eAppendix in Supplement 1). Mediation models were conducted with the mediation package in R. Because this package does not support complex nested designs, a random intercept was allowed only for family; site was included as a fixed effect. To test the moderating association of gender diversity with the association between being bullied and PLEs, an interaction term between being bullied and gender diversity was included. Longitudinal analyses allowed the coefficient of the main independent variable to vary by time within subject, within family, and within site (ie, random slope by time). Additionally, to test whether change in PLE scores over time differed by gender diversity (main association: most vs least gender diverse) and/or state policy, interaction terms between the variables of interest and time were included. All models were repeated with broad mental health problems as the dependent variable to test whether any observed significant associations were specific to PLEs. All reported cross-sectional models included age, pubertal development, birth-assigned sex, parental educational level, and combined family income as fixed-effects covariates. Supplementary analyses included race and ethnicity as covariates^31,32^ (see eMethods in Supplement 1). Race and ethnicity categories included American Indian, Alaska Native, or non-Hispanic Pacific Islander; Asian; Black; White; multiracial; other race (no additional information given); and unknown or not reported. Longitudinal models allowed a random slope by time and included the same covariates but omitted age because it was collinear with time. Models examining associations of state policies covaried for a state’s Gini coefficient, a time-variant measure of state income inequality (see eMethods in Supplement 1 for details about covariates). A 2-sided *P* < .05 was considered statistically significant.

## Results

In this sample of 8463 youths (mean [SD] age, 12.9 [0.6] years; 4490 [53.1%] assigned male at birth and 3973 [46.9%] assigned female at birth; 50 [0.6%] American Indian, Alaska Native, or non-Hispanic Pacific Islander, 179 [2.1%] Asian, 1018 [12.0%] Black, 5800 [68.5%] White, 1028 [12.1%] multiracial, 312 [3.7%] other race, and 76 [0.9%] unknown or not reported), 531 were categorized into the most gender-diverse group based on their responses on the felt-gender survey. The most gender-diverse group was overrepresented for those assigned female at birth (440 [82.9%], *P* < .001) (Table). Participants in this group scored 0.78 SD higher on the measure of PLEs (PQ-BC) than their least gender-diverse peers (β = 0.78; 95% CI, 0.69-0.86; *P* < 001) (eTables 4 and 5 in Supplement ). Additionally, the most gender-diverse group endorsed more broad mental health problems (BPM; β = 0.90; 95% CI, 0.80-0.99; *P* < 001) (eTable 6 in Supplement 1) and being bullied (PEQ-Vic; β = 0.48; 95% CI, 0.39-0.56; *P* < .001) (eTables 7 and 8 in Supplement 1); the latter finding remained significant after controlling for bullying perpetration (β = 0.35; 95% CI, 0.28-0.43; *P* < .001) (eTable 9 in Supplement 1).

### Mediating Association of Being Bullied

**Figure 3.**
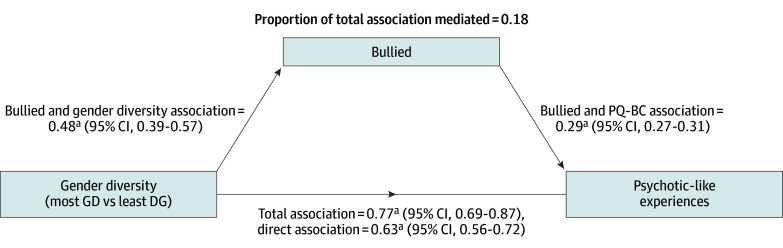
Path Diagram of the Mediating Association of Being Bullied With the Association Between Gender Diversity and Psychotic-Like Experiences (Prodromal Questionnaire–Brief Child Version [PQ-BC]) Each model contained age, birth-assigned sex, pubertal development, parental educational level, family income, and site as fixed effects and family identification as a random intercept. GD indicates gender diverse. ^a^*P* < .005.

In the mediation analysis, the indirect association of gender diversity with PLEs through being bullied was significant, with 18% of the total association mediated by being bullied (indirect association = 0.15, *P* < .001; direct association = 0.63, *P* < .001) (Figure 3). Results from sensitivity analyses robust to the zero-inflated distribution of the PQ-BC (ie, hurdle models) were largely consistent (eTables 10 and 11 in Supplement 1). Likewise, the difference in broad mental health problems by gender diversity was partially mediated by being bullied (16% mediated; indirect association = 0.15, *P* < .001; direct association = 0.75; *P* <.001) (eFigure 4 in the Supplement).

### Differential Associations of Being Bullied With PLEs

Across all participants, being bullied was associated with significantly greater PLE scores (β = 0.32; 95% CI, 0.30-0.34; *P* < .001) (eTables 12 and 13 in Supplement 1). In linear models, the association of being bullied with PLEs was significantly stronger in the most gender-diverse group compared with that in the least gender-diverse group (β = 0.18; 95% CI, 0.12-0.25; *P* < .001) (Figure 3; eTable 14 in Supplement 1). However, the interaction effect was not significant in the conditional model of the hurdle models (eTable 15 in Supplement 1). Greater instances of being bullied were associated with higher broad mental health problem scores across all participants (β = 0.39; 95% CI, 0.36-0.41; *P* < .001) (eTable 16 in Supplement 1), but there was no significant interaction effect with gender diversity (β = 0.07; 95% CI, −0.001 to 0.14; *P* = .052) (eTable 17 in Supplement 1), indicating similar association of bullying with general psychopathology regardless of gender diversity.

### Comparison of Cross-Sectional Associations in States With High and Low Support Policies

PLEs did not differ in states with high vs low supportive gender identity–related laws at a single time point (eTable 18 in Supplement 1). In the overall sample, regardless of gender diversity, being bullied was significantly lower in states with high vs low gender identity support (β = −0.09; 95% CI, −0.16 to −0.02; *P* = .02) (eTable 18 in Supplement 1). However, post hoc analyses revealed that self-reported bullying did not differ significantly by policy support in the most gender-diverse group compared with the least gender-diverse group (eTable 18 in Supplement 1). The association of being bullied with PLEs did not differ between the most gender-diverse group in high support states compared with the least gender-diverse group in low support states (β = −0.09; 95% CI, −0.22 to 0.04; *P* = .18) (eTable 19 in Supplement 1) or compared with the most gender-diverse group in low support states (β = −0.04; 95% CI, −0.25 to 0.17; *P* = .69) (eTable 20 in Supplement 1).

### Linear Changes in PLEs Over Time and Association With State Policies

**Figure 4.**
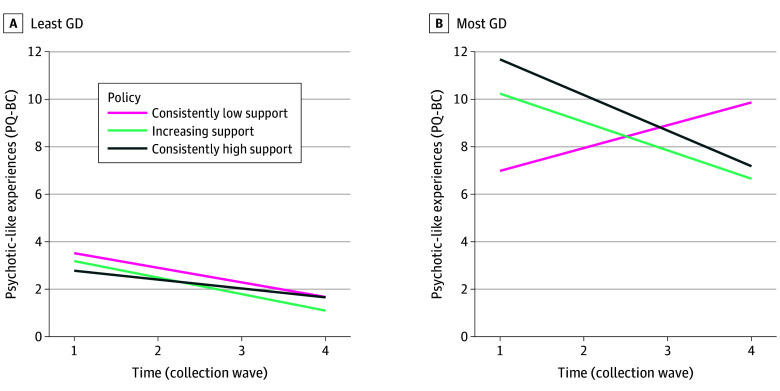
Line Graph of the 3-Way Interaction Among Time, State-Level Policy, and Gender Diversity Plotted are the projected values from the models regressing Prodromal Questionnaire–Brief Child Version (PQ-BC) values on the interaction term for time, state-level policy, and gender diversity along with covariates (birth-assigned sex, puberty levels, parental educational level, and family income), with slope over time allowed to vary within participants nested within family within site. A regression line with a positive slope is interpreted as an increase in PQ-BC over time. For reference, in the 3-way interaction model, the slope of each of these lines is compared statistically to the black line in the left panel (ie, PQ-BC change over time in the least gender-diverse [GD] group in consistently supportive states). Only the least GD group in states with consistently unsupportive policies shows a significantly different change (increase) in PQ-BC scores over time. See eTable 19 in Supplement 1 for accompanying statistics (reported in standardized coefficients).

Across years 1 through 4, corresponding to mean ages of 10.9 through 14.1 years, PLE scores decreased by 0.10 SD per year (β = −0.10; 95% CI, −0.13 to −0.08; *P* < .001) (eTable 21 in Supplement 1); that is, PLEs were less frequently endorsed over time in the whole sample. This longitudinal change in PLEs varied by gender-diverse group such that, compared with the least gender-diverse group, PLEs decreased more slowly in the most gender-diverse group (β = 0.06; 95% CI, 0.02-0.10; *P* = .002) (eTable 22 in Supplement 1). In states that consistently lacked supportive gender identity policies, PLEs increased over time in the most gender-diverse youths compared with the least gender-diverse youths residing in consistently supportive states (3-way interaction β = 0.31; 95% CI, 0.21-0.42; *P* < .001) (Figure 4; eTable 23 and eFigure 2 in Supplement 1). This 3-way interaction was robust to several sensitivity analyses, including hurdle models (eTables 24 and 25 and eResults in Supplement 1). Post hoc analyses revealed that within the most gender-diverse group, PLEs increased over time in consistently low support states compared with consistently high support states (eResults and eTables 26 and 27 in Supplement 1). This 3-way interaction was specific to PLEs and did not generalize to broad psychopathology, as measured by the BPM (β = 0.07; 95% CI, −0.05 to 0.19; *P* = .26) (eTable 28 in Supplement 1).

## Discussion

Our analysis of selected distal stressors that contribute to self-reported PLEs in youths recruited across 17 states, both at a single time point and over time, revealed several key findings with implications for clinical practice and policymaking. First, self-reported experiences of being bullied mediated the association between gender diversity and PLEs. That is, more gender-diverse youths endorsed more PLEs, a difference that was partially explained by increased reports of bullying. Second, unsupportive state policies exhibited nuanced associations between being bullied and PLEs: although there were no detectable associations of state policy at a single time point, more gender-diverse youths exhibited increases in PLEs over time in states that consistently lacked supportive legislation related to gender identity. In other states, regardless of gender diversity, the number of PLEs decreased or showed no change.

Our findings highlight the importance of considering both the immediate social environment and broader sociopolitical milieu when delivering care to gender-diverse youths and implementing interventions. Other data from the ABCD Study have suggested that stressful school environments contributed to broad mental health problems in gender-diverse adolescents.^33^ Antibullying interventions in schools have been found to be cost-effective,^34^ reduce bullying, and improve mental health among students.^35^ PLEs in youths are a risk factor for the development of chronic psychotic disorders, such as schizophrenia,^36^ which has an estimated annual economic burden of $343.2 billion in the US.^37^ Furthermore, PLEs are associated with risk for later poor functional outcomes and mental health problems, including suicidal behaviors.^38,39^ Thus, antibullying measures may help promote well-being and prevent the onset of serious mental illness in gender-diverse young people. Additionally, given the role of interpersonal stressors in the occurrence of PLEs in gender-diverse youths and the potential for misdiagnosis of psychosis,^8^ it is critical for clinicians to consider gender minority stress in assessment and treatment planning.^10^ Of note, although it was not the focus of the current study, future studies may interrogate differential effects of being bullied and state policies related to gender identity on individuals assigned male or female at birth, given the large discrepancy in birth-assigned sex observed within the most gender-diverse group in the current sample.

Findings suggest that long-term exposure to unsupportive legislation may exert negative effects on PLEs over time in gender-diverse youths. The cross-sectional findings indicating no differences by state policy in PLEs, being bullied, or their association were somewhat unexpected. Prior cross-sectional studies among TGD adults have shown that awareness of local antitransgender laws was associated with mental health problems^40,41^ and that TGD adults in areas lacking protective laws reported more frequent exposure to discriminatory experiences.^42^ The current study differs notably in that the sample consisted of youths aged 9 to 13 years who did not necessarily self-identify as TGD. Additionally, youths may have less awareness of state-level policy compared with TGD adults. Despite the unexpected cross-sectional findings, longitudinal findings were aligned with our hypotheses and the minority stress framework, which describes the association of minority stress with mental health as a chronic process.^12^ Indeed, a recent study^20^ showed that the passing of antitransgender legislation had the largest association with suicidal behaviors 1 to 2 years later. The current data show that in states with consistently unsupportive policies, PLEs increased in more gender-diverse teens, whereas in all other groups PLEs decreased or remained stable.

Our findings suggested that long-term exposure to unsupportive political environments has a somewhat specific, harmful effect on PLEs. This effect was not observed with a measurement of broad mental health problems, which includes measurement of both internalizing (eg, anxiety, depression) and externalizing (eg, attention, conduct problems) problems. Importantly, this difference does not imply that unsupportive policy has no effect on all other types of mental health difficulties. Our research questions were focused on PLEs and used a coarse measurement of general psychopathology as a comparison; thus, this study was not designed to make comprehensive inferences about the far-reaching impacts of unsupportive policy. The data presented here are consistent with theory and observation in other minoritized groups, in which stigma has been associated with increased hypervigilance^43^ and clinically significant paranoia,^23^ a central^22^ and perpetuating^44^ feature of psychosis. Thus, the long-term presence of unsupportive policy may contribute to hypervigilance in teens exploring gender, which in turn is captured via self-reported PLEs and perpetuates a cycle of worsening psychosis spectrum symptoms.

### Limitations

Several limitations of the current study should be considered. As aforementioned, the nature of the policy measurement used precludes the investigation of the effects of specific policies. Additionally, the state-level policy variables provided by MAP represent the legislative climate at the start of each year in which a participant’s data were collected (January 1). This timing limited the ability to assess specific temporal dynamics of policy effects on mental health. Furthermore, although state-level income inequality was controlled for, there may be other state-level characteristics that are associated with gender identity–related legislation and influence. Additionally, participants’ states of residence were inferred from data collection sites rather than residential addresses, which are not publicly available.

## Conclusions

This study adds to the sparse literature on the association of state policy with PLEs in gender-diverse adolescents, using a dimensional measure of gender independent of gender identity. The results suggest that (1) regardless of self-endorsed gender identity, the mental health of youths with dimensionally incongruent experiences of gender is negatively associated with mechanisms similar to those impacting TGD-identifying individuals; (2) PLEs in the context of gender diversity are exacerbated by bullying; and (3) gender diversity is only associated with the progression of PLEs in the context of consistently unsupportive state policies. These data suggest that continued sociopolitical stigma against gender diversity may contribute to higher rates of PLEs in youths and emphasize the need for antibullying interventions and careful consideration of legislation that perpetuates unsupportive state policies.

## Notes

Supplement 1. **eMethods.** Supplementary methods **eResults.** Supplementary results **eFigure 1. **Distribution of policy proportional difference scores **eFigure 2. **Depiction of the influence of baseline participation year on the derivation of the state policy variable **eFigure 3. **Map of the United States showing the Gender Identity Tally proportion variable across time in the ABCD sample **eFigure 4. **Path diagram representing the mediating effect of bullying victimization on the relationship between gender diversity and broad mental health problems (BPM total) **eFigure 5. **Representation of three-way interaction between time, longitudinal policy, and gender diversity group **eTable 1. **Gender diversity groupings are partially overlapping with, yet independent from, gender identity **eTable 2. **From the Movement Advancement Project website providing an example of two laws that would receive a value of -1 vs -0.5 (top; unsupportive laws) and 1 vs 0.5 (bottom; supportive laws) **eTable 3. **Data omitted from analyses were statistically significantly different across multiple variables of interest **eTable 4. **Participants in the most gender diverse group report greater psychotic-like experiences (PQ-BC) **eTable 5. **Participants in the most gender diverse group report greater psychotic-like experiences (PQ-BC), with race and ethnicity as possible effect modifiers **eTable 6. **Participants in the most gender diverse group report greater broad mental health problems (BPM total) **eTable 7. **Participants in the most gender diverse group report more frequent experiences of bullying victimization (PEQ-Victimization) **eTable 8. **Participants in the most gender diverse group report more frequent experiences of bullying victimization (PEQ-Victimization) with race and ethnicity as possible effect modifiers **eTable 9. **Participants in the most gender diverse group report more frequent experiences of bullying victimization (PEQ-Victimization) after controlling for experiences of bullying perpetration (PEQ Perpetration) **eTable 10. **In hurdle models, the total effect of gender diversity on PLEs (PQ-BC) is attenuated relative to results from linear models; however, the term remains significant **eTable 11. **In hurdle models, the direct effect (*c’*) of gender diversity on PQ-BC is attenuated when accounting for PEQ Victimization (*b*) **eTable 12. **Regardless of gender diversity, experiences of bullying victimization (PEQ Victimization) are associated with psychotic-like experiences (PQ-BC) **eTable 13. **Regardless of gender diversity, experiences of bullying victimization (PEQ Victimization) are associated with psychotic-like experiences (PQ-BC) with race and ethnicity as possible effect modifiers **eTable 14. **In linear models, the effect of bullying victimization (PEQ-Victimization) on psychotic-like experiences (PQ-BC) is larger in the most gender diverse compared to least gender diverse group (PEQ Victimization x Gender diversity (Most GD) term) **eTable 15. **In hurdle models, the interaction effect described in Supplementary Table 14 was attenuated and no longer significant **eTable 16. **Regardless of gender diversity, experiences of bullying victimization (PEQ Victimization) are associated with broad mental health problems (BPM total) **eTable 17. **In the most compared to least gender diverse group, there is no difference in the effect of bullying victimization (PEQ-Victimization) on broad mental health (BPM total; see PEQ Victimization x Felt gender (Most GD) term) **eTable 18.** Reported bullying victimization was lower in states with high versus low support at a single timepoint; otherwise, psychotic-like experiences (PQ-BC) and bullying victimization (PEQ-Vic), particularly within Most GD participants, did not differ by state policy support **eTable 19. **The effect of bullying victimization (PEQ-Victimization) on psychotic-like experiences (PQ-BC) is weaker in the most gender diverse participants in states with supportive policies **eTable 20. **The effect of bullying (PEQ Victimization) on psychotic-like experiences (PQ-BC) is no different in the most gender diverse participants in low-support states compared to the most gender diverse participants in high-support states **eTable 21. **On average, across the entire sample, rates of psychotic-like experiences (PQ-BC) declined over time **eTable 22. **Regardless of state policies at a single time point, PLEs declined more slowly in the most gender diverse participants (Most GD) **eTable 23. **Psychotic-like experiences differentially increased over time in the most gender diverse participants in consistently low support states **eTable 24. **Psychotic-like experiences differentially increased over time in the most gender diverse participants in consistently low support states – including data from Years 1 through 3 **eTable 25. **Psychotic-like experiences differentially increased over time in the most gender diverse participants in consistently low support states – Hurdle sensitivity model **eTable 26. **In the Most GD group in states with consistently unsupportive policies related to gender identity, the change in PQ-BC over time was greater than in the Most GD participants in states with consistently supportive policies **eTable 27. **Psychotic-like experiences show significant changes over time only in the most gender diverse group in states with consistently unsupportive policy. **eTable 28. **Broad mental health problems do not change differentially over time in the most gender diverse participants in consistently low support states **eAppendix.** Equations
eReferences

Supplement 2.
Data Sharing Statement
