# Supplementary material for: Bullying, State Policy, and Mental Health Symptoms in Gender-Diverse Youths
Source: JAMA Netw Open. 2026 Apr 21;9(4):e268104. doi: 10.1001/jamanetworkopen.2026.8104 (PMC13100838; doi:10.1001/jamanetworkopen.2026.8104)
Supplement: Supplement 2. — Data Sharing Statement [file jamanetwopen-e268104-s002.pdf]

Data Sharing Statement

Hughes. Bullying, State Policy, and Mental Health Symptoms in Gender-Diverse Youths. *JAMA Netw Open*. Published April 21, 2026. doi:10.1001/jamanetworkopen.2026.8104

Data
Data available: No

Additional Information
Explanation for why data not available: ABCD data are available here: https://www.nbdc-datahub.org/. Current data from the Movement Advancement Project can be found here: https://www.lgbtmap.org/equality-maps; historical MAP data used in the current study must be requested directly from MAP. Code to reproduce results is made available on GitHub (https://github.com/hughesdy). The data needed to reproduce results include raw ABCD data, accessed via NBDC which requires permission from ABCD and NIH, and derivatives of MAP data, which are made available on GitHub. Raw MAP data can be accessed via request to MAP.
